# First aid guidelines for psychosis in Asian countries: A Delphi consensus study

**DOI:** 10.1186/1752-4458-2-2

**Published:** 2008-02-21

**Authors:** Anthony F Jorm, Harry Minas, Robyn L Langlands, Claire M Kelly

**Affiliations:** 1 ORYGEN Research Centre, Department of Psychiatry, University of Melbourne, Locked Bag 10, Parkville, Victoria 3031, Australia; 2 Centre for International Mental Health, School of Population Health, University of Melbourne, Parkville, Victoria 3010, Australia

## Abstract

**Background:**

Guidelines for how a member of the public should give first aid to a person who is becoming psychotic have been developed for English-speaking countries. However, these guidelines may not be appropriate for use in other cultures. A study was therefore carried out to examine whether it was possible to achieve consensus on guidelines that could apply in a range of Asian countries.

**Methods:**

A Delphi consensus study was carried out with a panel of 28 Asian mental health clinicians drawn from Cambodia, China, Hong Kong, Indonesia, Japan, Malaysia, Mongolia, South Korea, Sri Lanka, Taiwan, Thailand and Vietnam. The panel was given a 211 item questionnaire about possible first aid actions and asked to rate whether they thought these should be included in guidelines. Panel members were invited to propose additional items.

**Results:**

After three Delphi rounds, there were 128 items that were rated as "essential" or "important" by 80% or more of the panel members. These items covered: recognition of psychosis, encouraging and assisting the person to seek help, how to interact with the person, responding to acute psychosis, responding to aggression, and what to do if the person refuses to get professional help.

**Conclusion:**

Despite the diversity of the countries involved, there was consensus on a core set of first aid items that were considered as suitable for assisting a psychotic person. Future work is needed to develop guidelines for specific countries.

## Background

The concept of first aid for physical injuries and health crises is now familiar throughout the world. Many members of the public receive first aid training as part of their work role or see it as a citizen's duty to be able to assist others. However, the extension of this concept to mental disorders and crises has only recently begun. Mental health first aid can be defined as the help provided to a person who is developing a mental health problem or is in a mental health crisis. The first aid is given until appropriate professional help is received or until the crisis resolves.

A Mental Health First Aid training course has been developed in Australia and has spread to other countries (Canada, England, Finland, Hong Kong, Ireland, Scotland, Singapore, USA, Wales) [1]. This training course has been evaluated in an uncontrolled trial [2], two randomized controlled trials [3,4] and a qualitative study [5] and found to increase the amount of assistance that first aiders give to others.

While this training appears to be effective, it may not be optimal in what it teaches. There is limited evidence available about what is the best course of action to assist people developing mental disorders or in mental health crisis situations. In conventional physical first aid there are national standards to guide what should be taught. In Australia, for example, these are developed by the Australian Resuscitation Council [6]. However, there have not been similar guidelines for mental health first aid situations. To fill this gap, a series of Delphi consensus studies is being carried out using expert panels of clinicians, consumers and carers drawn from developed English-speaking countries. The results of these consensus studies have been published on first aid for depression [7] and psychosis [8], and there will be future consensus guidelines on other situations, including helping a suicidal person and one who is engaging in deliberate self-injury.

Widening the base of people with some knowledge and skills in helping people with mental health problems is likely to result in many benefits [5]. In most low and middle income countries, where there is very little mental health system investment and grossly inadequate mental health workforce and service infrastructure [9], the treatment gap is very wide [10]. In such circumstances it is vital to engage an informed and capable general community in recognizing and responding to mental health problems and mental health crises. Mental health first aid training for community members can provide valuable support to the small and unevenly distributed professional mental health workforce.

However, while the guidelines that have been developed for English-speaking countries are suitable for the countries where the panel members came from, they may not be applicable for countries with very different cultures or health systems, or for cultural minorities within English-speaking countries. Because mental health first aid guidelines may not be culturally transportable, we have carried out an exploratory Delphi study on first aid for psychosis in Asian countries. The expert panel was drawn from clinicians in a range of Asian countries who were fluent in English. While we recognize the diversity of the countries involved, we wished to examine whether a consensus process was feasible and whether there were some first aid principles that might be broadly applicable. Psychosis was chosen as the topic because psychotic disorders are the major priority for mental healthcare systems in these countries.

## Methods

### The Delphi process

The general features of the Delphi methodology have been described in the literature [11]. There are many variants, but all involve a group of experts making private ratings of agreement with a series of statements, feedback to the group of a statistical summary of the ratings, and then another round of rating. Delphi group members do not meet, so it is possible to do studies using mail or the internet. The output from the process is statements for which there is substantial consensus in ratings.

### Expert panel

Panel members were drawn from the graduates of the International Mental Health Leadership Program [12]. This is an initiative of The Centre for International Mental Health, University of Melbourne, and The Department of Social Medicine, Harvard Medical School. It is a leadership training program and an international network of mental health professionals committed to mental health system development. Questionnaires were sent to 59 mental health clinicians who had trained in the program. Responses were received from 28 from the following countries and territories: Cambodia, (n = 1), China (n = 5), Hong Kong (n = 1), Indonesia (n = 5), Japan (n = 3), Malaysia (n = 1), Mongolia (n = 1), Sri Lanka (n = 1), South Korea (n = 3), Taiwan (n = 1), Thailand (n = 2) and Vietnam (n = 4). All panel members were medically qualified and most were psychiatrists. The panel comprised 20 males and 8 females. The ages of the panel members were: 13 aged 30–39 years, 13 aged 40–49 years, and 2 aged 50–59 years. Information was not collected on the clinical experience of the panel members.

### Development and administration of the questionnaire

The method of developing the questionnaire has been described previously [8]. Briefly, the content was based on a systematic search of websites, books, carer and consumer manuals, and journal articles for statements about how to help someone who may be experiencing a psychotic episode. These statements were grouped based on their common themes and used by a working group to generate questionnaire items specifying what actions a first aider should take. No judgements were made by the working group about the potential usefulness of the statements. Anything was included that fitted the definition of first aid, even if contradictory to other statements. The questionnaire was organized into sections of items on a common theme. These sections covered: recognizing and acknowledging that someone may be experiencing psychosis, encouraging the person to seek help, helping the person, interacting with the person, how to respond if the person becomes acutely psychotic, how to respond if the person becomes aggressive, and how to respond if the person denies they are unwell and/or refuses to get help.

The questionnaire was preceded by the following instructions: "Please complete the questionnaire by rating each statement according to how important you believe it is as a potential standard for Mental Health First Aid for psychosis. Please keep in mind that the standards will be used by the general public and as such, the statements need to be rated according to how important each one is as a way for someone, who does not necessarily have a medical or clinical background, to help a person who may have psychosis." Each statement was rated on the following scale: Essential, Important, Don't know/depends, Unimportant, Should not be included.

The questionnaire was administered as a web survey using the SurveyMaker application (surveymaker.com.au), with the option to complete it by email or paper mail if this was not possible. In Round 1 of the survey, panel members were asked to respond to the initial 219 items and were given an open-ended question at the end of each section asking for comments. If the comments included any new first aid statements, these were used to develop additional items for next Delphi round.

An item was accepted for inclusion in the guidelines if at least 80% of panel members rated it as "essential" or "important". If 70–79% gave this rating, the item was included for re-rating in the next round, with feedback to panel members about what percentage rated it highly in the previous round. This process continued for three rounds, which gave the opportunity for any new items to be rated and, if necessary, re-rated.

Once the list of consensus items was complete, these were used to write a piece of continuous text incorporating the first aid statements. This text was sent back to panel members for final endorsement and any suggestions for improvements. The final version constituted the guidelines.

### Ethics

Ethics approval was obtained from the University of Melbourne Human Research Ethics Committee (Project No. 0605537).

## Results

There were 28 panel members (47.5% of the 59 clinicians who were invited to participate) involved in Round 1, 22 in Round 2 and 21 in Round 3. The size of the panel was within the range that is typical of Delphi consensus studies (15–60 members) [13].

**Figure 1:**
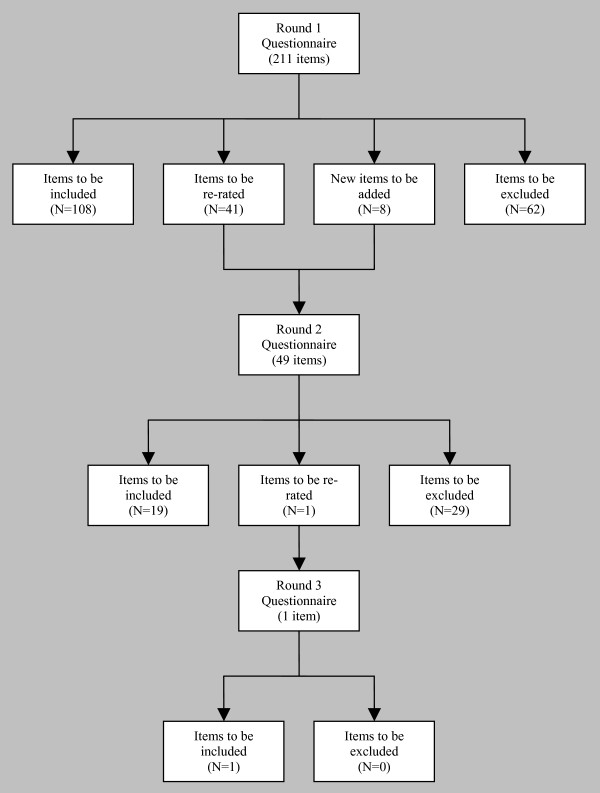
The number of items that were included, excluded and re-rated at each Delphi round.

Figure 1 shows what happened at each round in terms of inclusion or exclusion of items, and generation of new items. The Delphi process started with 211 items, 8 new items were written based on comments from panel members, and 128 met the 80%+ consensus criterion for inclusion in the guidelines.

Additional file 1 shows the items that were included, the round at which they achieved the criterion for inclusion and the percent consensus achieved. The final guideline statement is given in Additional file 2.

## Discussion

The findings show that it is possible to reach consensus about psychosis first aid despite the considerable diversity of cultures and health systems that the panel members came from. The resulting guidelines are a first step in providing guidance in Asian countries, but need to be followed up with more detailed studies in each country.

There are several differences between the guidelines for Asian countries and those for English-speaking countries. While all of the titles and section headings are the same, there are differences in which of the statements were accepted as first aid guidelines. Comparisons are difficult to make, because only professionals were involved in the consensus process in the Asian project, while consumers and carers participated in the development of the guidelines for English-speaking countries. A total of 49 items were accepted into the Asian guidelines for psychosis which were not accepted into the guidelines for English speaking countries. Of these, 9 were specific to the Asian panel, as they were suggested by panel members; 9 were endorsed by the professionals in the English-speaking panel and rejected by the consumers and carers, 5 were rejected by the consumers but endorsed by the other panel members, and 1 was endorsed by the carers and rejected by the other panel members. A further 5 items accepted by the Asian panel came very close to being accepted by the English-speaking panel. The remaining 20 items appear to represent real differences between the panels. The themes evident across these 20 items are that the Asian guidelines advise greater persistence in persuading the person to seek or accept professional help or hospitalisation, while they place a lesser emphasis on confidentiality.

The present study had a number of limitations. While the panel members came from diverse nations, the panellists were all medically trained and had all participated in a training program in Melbourne, so their views may reflect those of western psychiatry. This homogeneity of training may have increased consensus. Future studies should recruit broader and more representative expert panels including, where possible, professionals from all the relevant mental health disciplines and consumer and carer representatives. This is presently difficult in many Asian countries because there are very few (if any) clinical psychologists, psychiatric social workers, occupational therapists and mental health nurses. The participation of consumers and carers in such research – that is as members of an 'expert panel' rather than simply as research subjects – is uncommon.

Another limitation is that the inclusion of culturally relevant material was dependent on panellists writing in comments and few did this. This may have been, in most cases, due to lack of time. It may also be the observed phenomenon that, in questionnaires of all kinds that require ratings to be made, respondents rarely take the opportunity, when this is offered, to write comments or to make suggestions.

The questionnaire was administered in English rather than in the panellists' native languages. This of course limits the general applicability of the findings. It is possible that there would have been more cultural diversity in responses if panellists had used their native language. The next step in this work is to carry out detailed studies in each country. Such studies would differ from the work reported here in the following ways: the studies would be carried out in the language of the country and be managed by a local study coordinator; the expert panel would be more broadly representative, as indicated above; and, as well as the items identified in this work, potentially culturally relevant items (generated on the basis of a detailed knowledge of the local culture, particularly concerning culturally derived conceptions of mental health and illness [14,15] and culturally appropriate responses to mental health problems) would be included in the first round of the Delphi process.

## Conclusion

This exploratory study has demonstrated the utility of the Delphi process in reaching consensus about psychosis first aid across a number of Asian countries. The resulting guidelines (Additional file 2), similar to the guidelines developed in English-speaking countries, offer a first step in providing guidance in Asian countries. However, the process used to generate the guidelines is likely to have masked significant cultural differences across (and within) countries. Detailed studies within countries will be required to produce guidelines that are appropriate to specific cultural and mental health system contexts.

## Competing interests

The author(s) declare that they have no competing interests.

## Authors' contributions

AFJ originated the project, had input into the development of the questionnaire and co-wrote the manuscript. HM originated the project, provided the contacts with panel members and co-wrote the manuscript. RLL had the major role in developing the questionnaire, ran the Delphi study, analyzed the data and edited the manuscript. CMK had input into the development of the questionnaire, wrote the final guidelines and edited the manuscript. All authors read and approved the final manuscript.

## Supplementary Material

Additional file 1 Items rated as "essential" or "important" by at least 80% of panel members. Table of data showing the items included in the Delphi survey and the endorsement levels from the panel members. Click here for file

Additional file 2 Psychosis first aid guidelines for Asian countries. Text of guidelines written from the statements endorsed in the Delphi survey. Click here for file
