# Supplementary material for: First aid guidelines for psychosis in Asian countries: A Delphi consensus study
Source: Int J Ment Health Syst. 2008 Feb 21;2:2. doi: 10.1186/1752-4458-2-2 (PMC2278127; doi:10.1186/1752-4458-2-2)
Supplement: Additional file 1 — Items rated as "essential" or "important" by at least 80% of panel members. Table of data showing the items included in the Delphi survey and the endorsement levels from the panel members. [file 1752-4458-2-2-S1.doc]

## Items rated as “essential” or “important” by at least 80% of panel members

| **Item** | **Round in which item accepted** | **Percent endorsement in that round** |
| --- | --- | --- |
| The first aider should be able to recognise the early warning signs and/or symptoms of psychosis. | 1 | 100 |
| The first aider should realize that although warning signs and/or symptoms of psychosis are often not very dramatic on their own, taken together they may suggest that something is not quite right. | 1 | 85.7 |
| The first aider should not ignore or dismiss warning signs and/or symptoms if they appear gradually and are unclear. | 1 | 85.7 |
| The first aider should not assume that the person exhibiting warning signs and/or symptoms is just going through a phase or misusing substances. | 1 | 85.7 |
| The first aider should be aware that the warning signs and/or symptoms of psychosis may vary from person to person and can change over time. | 1 | 96.4 |
| The first aider should take into consideration the spiritual and/or cultural context of the person’s behaviours. | 1 | 82.1 |
| People developing a psychotic disorder will often not reach out for help. If the first aider is concerned about someone, they should approach the person in a caring and non-judgemental manner to discuss their concerns. | 1 | 92.9 |
| The first aider should pay attention to the person’s emotional state and approach the person when they are as calm and as clear thinking as they can be. | 1 | 82.1 |
| The first aider should try to find some common ground for discussion, gradually building up towards more specific questions about the person’s psychotic experiences. | 1 | 85.7 |
| Someone who is experiencing profound and frightening changes such as psychotic symptoms will often try to keep them a secret. The first aider should be aware that the person they are trying to help might not trust them or might be afraid of being perceived as “different”, and therefore may not be open with them. | 1 | 89.3 |
| The first aider should state, in specific behavioural terms, why s/he is concerned about the person and should not speculate about their diagnosis. | 1 | 82.1 |
| The first aider should ask the person if they have felt this way before and if so, what they have done in the past that has been helpful. | 1 | 85.7 |
| The first aider should try to find out what type of assistance the person believes will help them. | 1 | 96.4 |
| The first aider needs to convey that early intervention is important to prevent symptoms from escalating, without putting pressure on the person to seek help. | 1 | 85.7 |
| The first aider should encourage the person seek appropriate professional help as soon as possible even if they are unsure whether the person is developing or experiencing psychosis. | 1 | 82.1 |
| The first aider should reassure the person that it’s okay to seek help and point out that seeking help is a sign of strength rather than a sign of weakness or failure. | 1 | 89.3 |
| The first aider should convey a message of hope to the person by assuring them that help is available and things can get better. | 1 | 92.9 |
| The first aider should inform the person that a thorough medical assessment is important as the person’s symptoms could signal a variety of conditions. | 1 | 82.1 |
| The first aider should know what services are available locally. | 1 | 96.4 |
| The first aider should try to determine whether the person has a supportive social network and if they do, the first aider should encourage them to utilise these supports. | 1 | 82.1 |
| The first aider should provide the person with information about local services. | 1 | 100 |
| If the person decides to seek professional help, the first aider should make sure that the person is supported both emotionally and practically in accessing services. | 1 | 85.7 |
| As far as possible, the first aider should get the permission of the person before writing or speaking to professionals or others about them. | 1 | 82.1 |
| The first aider should always treat the person with respect. | 1 | 89.3 |
| Unless the person is a danger to themselves or others, the first aider should respect their privacy and right to confidentiality. | 1 | 85.7 |
| The first aider should understand that the person may be behaving and talking differently due to psychotic symptoms. | 1 | 96.4 |
| The first aider must recognise that the person who may be experiencing psychosis may find it difficult to tell what is real from what is not real. | 1 | 96.4 |
| The first aider should avoid confronting the person and should not criticise or blame them. | 1 | 96.4 |
| The first aider should try to tailor their approach and interaction to the way the person is behaving (e.g. if the person is suspicious and is avoiding eye contact, the first aider should be sensitive to this and give the person the space they need). | 1 | 89.3 |
| The first aider should let the person know that s/he is there to support them. | 1 | 96.4 |
| The first aider should allow the person to talk about their experiences and beliefs if they want to. | 1 | 89.3 |
| The first aider should be honest when interacting with the person and should not make them any promises that cannot be kept. | 1 | 89.3 |
| The first aider should try to empathise with how the person feels about their beliefs and experiences, without stating any judgments about the content of those beliefs and experiences. | 1 | 82.1 |
| The first aider should avoid being intrusive as intense, interpersonal interactions can make psychotic symptoms worse. | 1 | 82.1 |
| The first aider should not use sarcasm when interacting with a person who may be experiencing psychosis. | 1 | 89.3 |
| The first aider should ask the person about what will help them to feel safe and in control. | 1 | 92.9 |
| If the person is unwilling to talk with the first aider, the first aider should not try to force them to talk about their experiences. | 1 | 92.9 |
| If the person is unwilling to talk with the first aider, the first aider should let them know that s/he will be available if they would like to talk in the future. | 1 | 92.9 |
| The first aider should recognise that the person may be frightened by their thoughts and feelings. | 1 | 92.9 |
| If the person is very fearful, the first aider should keep them company to reassure them that they are not alone. | 1 | 85.7 |
| The first aider should reassure the person that s/he is there to help the person and wants to keep them safe. | 1 | 89.3 |
| The first aider should recognise that the delusions and/or hallucinations are very real to the person. | 1 | 92.9 |
| The first aider should not dismiss, minimise or argue with the person about their delusions and/or hallucinations. | 1 | 96.4 |
| The first aider should not use direct psychiatric terms, such as hallucination or delusion, when speaking to the person as these terms can imply immediate dismissal of the person’s ideas and experiences. | 1 | 85.7 |
| The first aider should not act alarmed, horrified or embarrassed by the person’s hallucinations or delusions. | 1 | 96.4 |
| The first aider should not laugh at the person’s symptoms of psychosis. | 1 | 100 |
| Until the first aider knows the content and context of the person’s delusions, it is important to keep themselves safe from potentially aggressive reactions. | 1 | 96.4 |
| The first aider should ask questions about the content of the person’s delusions, particularly any elements that indicate the potential for harming themselves or others. | 1 | 89.3 |
| If the person exhibits paranoid behaviour, the first aider should not encourage or inflame the person’s paranoia. | 1 | 96.4 |
| If the person is showing a limited range of feelings, the first aider should be aware that it does not mean that the person is not feeling anything. | 1 | 96.4 |
| The first aider should not assume the person cannot understand what they are saying, even if the person’s response is limited. | 1 | 89.3 |
| In the event of a crisis, when the person experiencing psychosis has become acutely unwell… |  |  |
| …the first aider should try to remain as calm as possible. | 1 | 92.9 |
| …the first aider should evaluate the situation by assessing the risks involved (e.g. whether there is any risk that the person will harm themselves or others). | 1 | 100 |
| …the first aider should assess whether the person is at risk of suicide. | 1 | 100 |
| …the first aider should assess whether it is safe for the person to be alone and if not, should ensure that someone stays with the person. | 1 | 100 |
| …the first aider should remember that they cannot reason with someone who is acutely psychotic. | 1 | 82.1 |
| …the first aider should be aware that the person might act upon a hallucination or delusion. | 1 | 100 |
| …the first aider should remember that their primary task is to de-escalate the situation and therefore should not do anything to further agitate the person. | 1 | 89.3 |
| …the first aider should try to maintain safety and protect the person, themselves and others around them from harm. | 1 | 100 |
| …the first aider should allow as safe a physical distance between themselves and the person as practical to maintain interaction. | 1 | 82.1 |
| …the first aider should have access to an exit. | 1 | 85.7 |
| …the first aider should ask the person whether they would like her/him to decrease distractions (e.g. turn off TV, radio, dishwasher etc.) and lower stimulation (e.g. reduce room lights). |  | 85.7 |
| …the first aider should communicate in a clear and concise manner and use short, simple sentences. | 1 | 92.9 |
| …the first aider should try to find out if the person has anyone s/he still trusts (e.g. close friends, family) and should try to enlist their help. | 1 | 92.9 |
| …the first aider should remain aware that they may not be able to de-escalate the situation and if this is the case, they should be prepared to call for assistance. | 1 | 100 |
| …the first aider should explain to the person why they believe that a professional assessment is necessary. | 1 | 92.6 |
| …and the person is a danger to themselves or others, the first aider should make sure they are evaluated by a medical or mental health professional immediately. | 1 | 96.4 |
| …if other people arrive, the first aider should explain to the person experiencing psychosis who the people are, that they are there to help and how they are going to help. | 1 | 89.3 |
| …the first aider should try to limit access to objects that the person could use to harm themselves or others until crisis staff arrive. | 1 | 96.4 |
| …if crisis staff arrive, the first aider should convey specific, concise observations about the severity of the person’s behaviour and symptoms to the crisis staff. | 1 | 96.4 |
| …if hospitalisation is required and the person will not go voluntarily, the first aider should see if one of the person’s relatives or friends can persuade them to go to hospital. | 1 | 82.1 |
| …if the person needs to be hospitalised, the first aider should support them by focusing conversation on how a hospital stay will bring relief through reducing psychotic symptoms. | 1 | 82.1 |
| …if the first aider is a family member or significant other, they should be prepared to seek involuntary hospitalisation for the person if necessary. | 1 | 82.1 |
| The first aider should be aware that people with psychosis are not usually aggressive and are at a much higher risk of harming themselves than others. | 1 | 89.3 |
| The first aider needs to recognise that certain symptoms of psychosis (e.g. visual or auditory hallucinations) can cause people to become aggressive. | 1 | 100 |
| The first aider should know how to de-escalate the situation if the person they are trying to help becomes aggressive. | 1 | 100 |
| If necessary, the first aider should remove any weapons or objects that could be used as weapons from the person’s immediate environment. | 1 | 92.9 |
| If the person is showing aggression, the first aider should avoid raising their voice and should not talk too fast. | 1 | 92.9 |
| If the person is showing aggression, the first aider should stay calm and avoid nervous behaviour (e.g. shuffling their feet, fidgeting, making abrupt movements). | 1 | 100 |
| The first aider should not respond in a hostile, disciplinary or challenging manner to the person who is being aggressive. | 1 | 96.4 |
| The first aider should avoid asking too many questions as they can spark defensiveness and further anger. | 1 | 96.4 |
| The first aider should not threaten the person as this may increase fear or prompt aggressive behaviour. | 1 | 92.9 |
| The first aider should remain aware that the person’s symptoms or fear causing the aggression may be exacerbated by the first aider taking certain steps (e.g. involving the police). | 1 | 85.7 |
| If the first aider is frightened, they should seek outside help immediately as they should never put themselves at risk. | 1 | 85.7 |
| If the police are called, the first aider should tell them that the person is experiencing a psychotic episode and that the first aider needs the help of the police to obtain medical treatment and to control the person’s aggressive behaviour. | 1 | 85.7 |
| The first aider should let the police know whether or not the person is armed. | 1 | 96.4 |
| If the first aider is alone with the person experiencing psychosis, they should contact someone to come and stay with them until professional help arrives. | 1 | 82.1 |
| The first aider should be aware that the person who is experiencing psychotic symptoms may lack insight that they are unwell. | 1 | 100 |
| If the person does lack insight, the first aider should be aware that they might actively resist the first aider’s attempts to encourage them to seek help. | 1 | 89.3 |
| When someone who is experiencing symptoms of psychosis denies that they are unwell, the first aider’s course of action should depend on the type and severity of the person’s symptoms. | 1 | 92.9 |
| The first aider should not try to convince the person that they are psychotic. | 1 | 92.9 |
| If the person refuses to seek help, the first aider should encourage them to talk to someone they trust. | 1 | 96.4 |
| The first aider should focus on trying to find something that the person agrees is a problem and then suggest that the person seek help for that (e.g. if the person says that they feel anxious around other people, the first aider should encourage them to seek help for anxiety). | 1 | 82.1 |
| The first aider should recognise that even if the person does realise that they are unwell, their confusion and fear about what is happening to them may lead them to deny that there is anything wrong. | 1 | 85.7 |
| A lot of people are hesitant to seek treatment for psychosis because they are afraid of being hospitalised. If possible, the first aider should reassure the person that if treatment is started early enough, hospitalisation might not be necessary. | 1 | 85.7 |
| The first aider should calmly express her/his worry to the person about their choice to not seek help and the potential implications. | 1 | 89.3 |
| The first aider should talk to the person’s family or close friends, as they may be able to facilitate the next step towards obtaining professional help for the person. | 1 | 92.9 |
| The first aider should provide a consistent message encouraging the person to seek help. | 1 | 89.3 |
| Because symptoms of mental illness may stem from other physical illnesses, the first aider should initially encourage the person to see their doctor for a check-up, rather than suggesting from the start that it may be psychosis. | 1 | 82.1 |
| If the person agrees to see a doctor for a check-up, the first aider should contact the doctor to let them know what is happening. | 1 | 85.7 |
| If the person refuses to get help, the first aider should remain friendly and open to the possibility that they may want the first aider’s help in the future. | 1 | 85.7 |
| The first aider should remain patient, as people experiencing psychosis often need time to develop insight regarding their illness. | 1 | 85.7 |
| The first aider should consult a mental health professional for advice on how to help the person. | 1 | 89.3 |
| If the first aider does discuss their concerns about the person with a doctor, they should make sure they clearly articulate their observations of the person who is experiencing psychotic symptoms (e.g. exactly what the person has been doing and saying, where and when) so that the doctor has all the necessary information. | 1 | 82.1 |
| If the person is acutely psychotic and denies that they are unwell, the first aider should contact the emergency services and ask for an assessment under the relevant mental health legislation. | 1 | 85.7 |
| If the person is taken to hospital, the first aider should ask to speak with the medical professional conducting the person’s assessment. The first aider will then be able to explain the symptoms that have been occurring as people experiencing psychosis who do not want treatment may hide their behaviour or ideas from a professional. | 1 | 89.3 |
| The first aider needs to recognise that unless a person with psychosis meets the criteria for involuntary committal procedures, they cannot be forced into treatment. | 1 | 89.3 |
| The first aider should never threaten the person with the mental health act or hospitalisation | 1 | 92.9 |
| The first aider should not assume that the warning signs and/or symptoms of psychosis will go away on their own. | 2 | 82.6 |
| The first aider should discuss the signs and/or symptoms of psychosis with the person in that person’s own language and in a way that is culturally meaningful to them. | 2 | 86.3 |
| The first aider should ask the person whether they have noticed changes in their behaviour. If they have noticed changes, the first aider should ask whether these changes are bothering them or whether they are distressed by their experiences. | 2 | 86.3 |
| If the person or their family do not think doctors can help with mental illnesses, the first aider should explain that the illness may be due to a problem in the brain which a doctor can treat. | 2 | 100 |
| If health professionals are available in the local community, the first aider should connect with these professionals so they can seek advice when needed. | 2 | 90.9 |
| The first aider should remain aware of the influence that the person’s family may have over them. For example, the family may help the person access appropriate professional care or they may prevent the person from obtaining the care that they need. | 2 | 95.4 |
| If either the person experiencing psychosis or the first aider lacks confidence in the medical advice they have received, they should seek a second opinion from another medical or mental health professional. | 2 | 81.8 |
| The first aider should remain aware of the person’s life situation and/or status in the community when they approach the person. | 2 | 81.9 |
| The first aider should ask the person if they want to talk about how they are feeling. | 2 | 90.9 |
| People experiencing symptoms of psychosis are often unable to think clearly. The first aider should respond to disorganized speech by communicating in an uncomplicated and succinct manner, and should repeat things if necessary. | 2 | 90.9 |
| After the first aider speaks, they should be patient and allow plenty of time for the person to digest the information and respond. | 2 | 81.8 |
| In the event of a crisis, when the person experiencing psychosis has become acutely unwell… |  |  |
| …if the person has an advance directive/relapse prevention plan, the first aider should follow the guidelines set out in the plan. | 2 | 81.8 |
| …if hospitalisation is required, the first aider should encourage the person to go voluntarily. | 2 | 81.8 |
| If the person’s aggression escalates out of control at any time, the first aider should remove themselves from the situation and call the crisis team if one is available. | 2 | 81.8 |
| When contacting the appropriate service, the first aider should not assume the person is experiencing a psychotic episode but should outline any symptoms and immediate concerns. | 2 | 86.4 |
| If the person is unwilling to seek help, the first aider should set aside some time to discuss their concerns with the person and back up any concerns with examples of behaviour or problems they have noticed. | 2 | 81.8 |
| If the person is unwilling to see a doctor or mental health professional, the first aider should explore the reasons why the person is unwilling to seek help. | 2 | 85.7 |
| The first aider should stress the potential benefits of getting help. | 2 | 95.5 |
| The first aider should assist by giving the person resources (e.g. pamphlets, telephone numbers for services). | 2 | 95.5 |
| The first aider should discuss the warning signs and/or symptoms with the person’s relatives | 3 | 92.5 |
