# Supplementary material for: First aid guidelines for psychosis in Asian countries: A Delphi consensus study
Source: Int J Ment Health Syst. 2008 Feb 21;2:2. doi: 10.1186/1752-4458-2-2 (PMC2278127; doi:10.1186/1752-4458-2-2)
Supplement: Additional file 2 — Psychosis first aid guidelines for Asian countries. Text of guidelines written from the statements endorsed in the Delphi survey. [file 1752-4458-2-2-S2.doc]

# Psychosis First Aid Guidelines for Asian Countries

How do I know if someone is experiencing psychosis?

You need to know and be able to recognise the symptoms of psychosis.

**Common symptoms when psychosis is developing^1^**

*Changes in emotion and motivation:*

Depression; anxiety; irritability; suspiciousness; blunted, flat or inappropriate emotion; change in appetite; reduced energy and motivation

*Changes in thinking and perception:*

Difficulties with concentration or attention; sense of alteration of self, others or outside world (e.g. feeling that self or others have changed or are acting differently in some way); odd ideas; unusual perceptual experiences (e.g. a reduction or greater intensity of smell, sound or colour)

*Changes in behaviour:*

Sleep disturbance; social isolation or withdrawal; reduced ability to carry out work or social roles

^1^Adapted from: Edwards, J & McGorry, PD (2002). *Implementing Early Intervention in Psychosis*. Martin Dunitz, London.

In **established psychosis** delusions and hallucinations are common.

A **delusion** is a false belief that cannot be altered by reason or contradictory evidence. The belief cannot be explained by the person's usual cultural and religious beliefs. A delusion is firmly maintained in the face of incontrovertible evidence that it is false. Delusions are a frequent feature of psychotic disorders.

A **hallucination** is a profound distortion in a person's perception of reality. A hallucination is a sensory experience (the person may see, hear, smell, taste, or feel something) in the absence of a sensory stimulus. The sensory experience is accompanied by a powerful sense of reality and has the qualities of a real perception.

Many of the experiences and behaviours that are common in psychosis may be subtle, particularly early in the development of psychotic disorder, and may not be dramatic on their own. However, when a number of these changes in experience and behaviour appear together, it suggests that something is not right. It is important that you don’t ignore such changes because they are unclear or they develop gradually. Experiences and behaviours commonly change over time, and they may be different in different people.

Don’t assume that the symptoms or the changes in behaviour are just part of a phase the person is going through, or that the person is abusing alcohol or other drugs. Also, you cannot assume that the symptoms will just go away on their own.

If you think that someone may be developing psychosis, you should discuss your concerns with them and their family, being careful to use language that they can understand. There may be relevant cultural and spiritual context that you will need to take into account.

How should I approach someone who may be experiencing psychosis?

When someone is experiencing psychosis, they will often not reach out for help. The experiences may be frightening or disturbing and they may try to hide them. You will need to approach the person in a caring and non-judgemental way to discuss your concerns. If possible, do this at a time when the person seems calm and is thinking clearly. The person may not trust you right away or may be concerned that they will be perceived as strange or different.

Begin by finding topics of discussion that will enable you to express your concerns and to ask specific questions about the person’s experiences. You should ask the person if they have noticed any changes in their thinking or feelings or behaviour and, if so, whether they are worried or distressed about these changes. Don’t speculate on the possible causes of these changes; instead, discuss the specific changes the person describes to you or that you have noticed.

Ask the person if they would like to talk about how they are feeling and then allow them to talk about their experiences, beliefs and perceptions if they wish to do so. However, if they do not wish to talk about these things do not insist that they do so. Rather, tell them that they can speak to you in the future if they wish to.

Ask the person if they have ever experienced anything similar before and, if so, what they found helpful at that time. Ask them as well what they think might be helpful for them now. Find out whether the person has a supportive social or family network and, if they do, encourage them to use these supports.

How can I be supportive?

You must always treat the person with respect. Keep in mind the person’s life situation and their place as a member of the community when talking to or approaching them. Your behaviour should be responsive to the person’s concerns or fears or behaviour; for example, if the person is feeling suspicious and does not make or maintain eye contact, do not stare at them. Avoid confronting the person and do not criticise or blame them for their symptoms or behaviour. Do not use sarcasm with a person who may be experiencing psychosis. It is important not to be intrusive - intense or confronting conversations can make psychotic symptoms worse.

Be aware that the person may talk or behave differently because of their symptoms and that they may not be able to distinguish what is real from what is not. Remember that they may be suspicious or fearful because of their thoughts and feelings, or because of their perceptions (for example they may be hearing threatening voices). You should reassure them that you will keep them company if this is the case. Empathise with how the person is feeling about their experiences, but do not state your judgements about the content of beliefs or thoughts or perceptions. Tell the person you are there to support them. Assure the person that you want them to be safe. Ask them what they think would help them to feel safer or more in control. Be honest and do not make any promises that cannot be kept.

Always remember that unless the person has become a danger to themselves or others you must respect their right to privacy and to confidentiality.

**How should I deal with communication difficulties?**

When experiencing symptoms of psychosis, people may have difficulty thinking clearly and may speak in a confusing and disorganised way. You should use clear, simple language and be prepared to repeat things if the person does not understand. Be patient and allow the person enough time to express what they wish to communicate and to understand and make sense of what you say. However, do not assume that the person has not understood you, even if their response is limited. Do not assume, either, that the person feels nothing if they are expressing only a limited range of feelings.

How do I deal with delusions and hallucinations?

If the person is experiencing delusions (false beliefs) or hallucinations (false perceptions) you must keep in mind that these are very real to the person who is experiencing them. Do not argue with the person about these, do not dismiss or minimise them, and do not try to persuade the person that their beliefs are wrong or that what they see or hear is not real. Do not laugh at the person or behave as though you are shocked or embarrassed by their experiences or by their behaviour.

You should ask about the content of the delusions and hallucinations, particularly anything that may indicate the potential that the person may harm themselves or others. Avoid using the terms ‘delusions’ and ‘hallucinations’ as these may lead the person to feel you are dismissing their experiences. Instead, ask questions such as: “what is happening?” and “what are you seeing and hearing right now?” Until you know the content of the delusions and hallucinations, it is important that you keep yourself safe from possible aggressive reactions. If the person seems suspicious or fearful, be careful not to do anything to encourage or inflame their suspicions or fears.

How do I encourage the person to get professional help?

Convey a clear message that help is available and things can get better. Reassure the person that it is okay to seek help and that it is a sign of strength, not weakness, to do so. Tell the person that getting help early is important, but be careful not to put too much pressure on them to do so. Even if you are not sure whether the person is experiencing psychosis, you should encourage the person to seek help as soon as possible. It may be that the person is not experiencing psychosis. Medical assessment will be able to determine this.

If the person is open to seeking help, you will need to be able to give the person information about mental health services that are available locally. If there are health professionals in the local community, you should connect with them so that you are able to get advice when you need it. However, in so far as it is possible, you should not discuss the person’s experiences or behaviour with health professionals without the person’s permission. If you or the person you are helping lack confidence in the health professional you speak to, seek a second opinion from a different professional.

You need to be aware of the influence that the person’s family might have over the person’s decisions regarding professional help. The family may be able to assist the person to obtain appropriate help or, in some cases, may try to prevent the person from obtaining the care they need. If the family does not believe that doctors can help with mental illnesses, discuss the availability of effective treatments in a way that is meaningful to the family. For example, you may explain that the illness may be caused by a problem in the brain that can be effectively treated with medicines.

Ensure that the person has both emotional and practical support to obtain professional help. Practical help may, for example, include transport to health services or taking care of children while the person attends appointments.

What should I do if the person doesn’t want help?

Set some time aside to discuss with the person what your concerns are and give them examples of their experience or behaviour that they have described as problematic or that you have noticed. Express your concern about their decision not to seek help and explain the possible consequences of this (for example, that their symptoms may get worse). Remember that even if the person is aware that they are unwell, they may deny that there is anything wrong or they may fear what will be done if they acknowledge their problems.

Try to find out why they do not want to seek professional help. Many people do not want to seek help for psychotic symptoms because they are afraid that they will be forced to go to hospital. If this is the case, reassure the person that if treatment is started early enough, hospital care may not be necessary.

Stress the potential benefits of getting help, such as relief from anxiety or frightening symptoms. Be consistent in encouraging the person to seek help and be patient; sometimes people start to develop awareness of their illness over time. Remain friendly, open and available to discuss the possibility of seeking professional help in the future. It might be helpful to provide the person with some written information about psychosis and phone numbers for services they may want to contact.

Two reasons that the person you are helping might refuse to accept professional help are: they may not be aware that their distressing experiences and behaviours are due to illness and that they need medical help, or they may be aware that they are unwell but simply do not want any professional help.

*If the person lacks awareness that they are ill:*

If the person is not aware that they are suffering from an illness or actively deny that they are ill, they are likely to resist your efforts to encourage them to accept professional medical help. Do not try to convince them that what they believe is not true or what they perceive (see or hear) is not real. Be guided by the type and severity of the person’s symptoms when deciding whether to seek help on their behalf.

*If the person is aware that help may be needed, but does not want it:*

It is often helpful to identify something that the person agrees is a problem, and encourage them to seek help for that. For example, if the person says they feel anxious around other people, you could encourage them to seek help for that. Sometimes the symptoms of psychosis may stem from other physical problems, so you may encourage the person to go to the doctor for a general check-up. If the person agrees to this, contact the doctor and explain what has been happening so that they are alert to the possibility of a psychotic illness. If the person agrees, it may be helpful for you to accompany them to a first appointment for medical assessment. The person may agree to having a doctor or nurse visit them at home for the assessment.

Encourage the person to talk to someone else that they trust. Consider talking to the person’s family members or close friends, as they may be able to take steps towards obtaining help for the person. You can also ask a health professional for advice on how to help the person. If you do this, you will need to clearly describe your observations of the person’s behaviour, such as what they have been doing and saying, and where and how often these symptoms occur, so the doctor has all the necessary information.

*In an emergency:*

If the person is acutely psychotic and denies that they are unwell, you will need to contact emergency services and ask for an assessment under the relevant mental health legislation. Be aware, though, that unless the person meets criteria for involuntary treatment (usually, the criteria include a clear threat to themselves or other people), they cannot be treated without their consent. Never threaten the person with involuntary hospitalisation.

If the person is taken to hospital, ask to speak with the medical professional conducting the assessment. Explain the symptoms that the person has described to you and the behaviour that you have observed. People experiencing psychosis who do not want treatment often conceal their thoughts, beliefs, feelings and behaviour from professionals.

What should I do if the psychotic symptoms are very severe?

First, you must evaluate the situation by assessing any risks, such as whether the person may be at risk of harm to themselves or to others. If the person you are helping is acutely unwell, it is very important that you stay as calm as possible.

Assess whether or not it is safe for the person to be left alone and, if not, make sure someone stays with them.

Always communicate using short, simple sentences and in a clear, concise manner. Ask the person if they would like you to do anything to decrease distractions (such as turning off the television or radio) or stimulation (for example, by turning down lighting).

Ask the person if there is someone they trust, such as close friends or family, and try to enlist their help. Keep in mind that it is not always possible to transform a tense or dangerous situation into one that is calm and safe. If you are unable to do so, you need to call for assistance. Explain to the person why you feel that assessment by a mental health professional is necessary.

When crisis staff, such as a mental health team or paramedics attend, explain to the person who they are and make sure that they explain what they are going to do to help. You need to explain to the crisis staff as precisely and specifically as possible what has been happening and how severe the symptoms have been.

The person may need hospital care. If this is the case, you should encourage the person to accept voluntary admission. If you are unable to persuade them, a family member or friend may be able to. Explain to the person that going to hospital will reduce their symptoms and bring relief. If they are frightened emphasise that they (and others, where appropriate) will be safe. If the person you are helping is a family member or spouse, you need to be prepared to seek involuntary hospitalisation for them if this becomes necessary.

What if the person becomes aggressive?

It is important to remember that people with psychosis are rarely aggressive and are at a much higher risk of harming themselves than harming others. From time to time, though, a person with psychosis may become aggressive. If this occurs, your main task is to ensure everyone’s safety and restore calm. Do not do anything that might further agitate the person or that will aggravate an already tense and potentially dangerous situation. Remember that when someone is acutely psychotic, it is not possible to reason with them.

Be aware that the person may act on the basis of delusions (for example the person may believe that you or someone else is about to attack them or otherwise harm them) or hallucinations (may be hearing voices that are compelling the person to do something). Do your best to maintain your own safety as well as the safety of the person and anyone else present. Make sure that you are a safe physical distance from the person and that you have access to an exit. If it seems that there is a risk of harm to anyone, you need to contact professional help right away. Limit the person’s access to anything that could be used to harm themselves or others until help arrives.

*Restoring calm and safety*

If the person with psychosis that you are helping is becoming aggressive, you need to know how to ensure safety and restore calm.

- Stay calm and avoid any nervous behaviour such as shuffling your feet or making any abrupt movements.
- Do not raise your voice or talk too quickly.
- Do not threaten the person, as this will increase their fear and may trigger an aggressive reaction.
- Avoid responding in a hostile, disciplinary or challenging manner.
- Do not ask a lot of questions as these can cause the person to become defensive, agitated or angry.

Remove any weapons, or items which could be used as weapons, from the immediate environment. Never put yourself at risk; if you are frightened, seek outside assistance immediately from the police or a mental health crisis services. If the person’s behaviour appears to be getting out of control, you must remove yourself from the situation and immediately call for help.

If it becomes necessary to call the police or mental health crisis services, be aware that this will usually further frighten the person and exacerbate their symptoms, but do not let this deter you from doing so when it is necessary. When you contact mental health crisis services or other health professionals, do not tell them that the person is experiencing psychosis as this can only be determined by a health professional. Instead, describe the person’s behaviour and your immediate concerns. Conversely, if you need to call the police, tell them that you believe the person may be having a psychotic episode, and that you need the police to help to control the person’s aggressive behaviour and obtain medical treatment for them. In addition, tell the police whether or not the person has a weapon.

If you are alone with the person, contact someone you trust to come and stay with you until professional help arrives.
